# Fire-derived organic matter retains ammonia through covalent bond formation

**DOI:** 10.1038/s41467-019-08401-z

**Published:** 2019-02-08

**Authors:** Rachel Hestrin, Dorisel Torres-Rojas, James J. Dynes, James M. Hook, Tom Z. Regier, Adam W. Gillespie, Ronald J. Smernik, Johannes Lehmann

**Affiliations:** 1 Soil and Crop Sciences, School of Integrative Plant Science, Bradfield Hall, Cornell University, Ithaca, NY 14853 USA; 2 Canadian Light Source Inc., 44 Innovation Boulevard, Saskatoon, SK S7N 2V3 Canada; 3 NMR Facility & Spectroscopy Lab, Mark Wainwright Analytical Centre and School of Chemistry, University of New South Wales, Sydney, NSW 2052 Australia; 4 Present Address: School of Agriculture, Food and Wine, The University of Adelaide, Waite Campus, Urrbrae, SA 5064 Australia; 5 Atkinson Center for a Sustainable Future, Rice Hall, Cornell University, Ithaca, NY 14853 USA; 6 School of Environmental Sciences, University of Guelph, Guelph, N1G 2W1 ON Canada

## Abstract

Fire-derived organic matter, often referred to as pyrogenic organic matter (PyOM), is present in the Earth’s soil, sediment, atmosphere, and water. We investigated interactions of PyOM with ammonia (NH_3_) gas, which makes up much of the Earth’s reactive nitrogen (N) pool. Here we show that PyOM’s NH_3_ retention capacity under ambient conditions can exceed 180 mg N g^−1^ PyOM–carbon, resulting in a material with a higher N content than any unprocessed plant material and most animal manures. As PyOM is weathered, NH_3_ retention increases sixfold, with more than half of the N retained through chemisorption rather than physisorption. Near-edge X-ray absorption fine structure and nuclear magnetic resonance spectroscopy reveal that a variety of covalent bonds form between NH_3_-N and PyOM, more than 10% of which contained heterocyclic structures. We estimate that through these mechanisms soil PyOM stocks could retain more than 600-fold annual NH_3_ emissions from agriculture, exerting an important control on global N cycling.

## Abstract (web summary)

Fire-derived organic matter (OM) is present throughout the environment, and its impact on nutrient cycling remains poorly understood. Here, the authors show that this pyrogenic OM can retain large quantities of ammonia through covalent bond formation, thereby exerting an important control on nitrogen cycling.

## Introduction

The Earth’s soil, atmosphere, marine sediment, and ocean water contain large quantities of pyrogenic C (54–109, 0.26 10^−3^, 480–1440, and 26–145 Pg of C, respectively^1,2^). In soil, most of this pyrogenic C originates from burnt biomass generated during vegetation fires, which contributes up to 129 Tg yr^−1^ of PyOM–carbon (PyOM–C) to soil C stocks^1^. Many aspects of pyrogenic C biogeochemistry remain poorly understood, including interactions between pyrogenic C’s heterogeneous surface—containing both aromatic and aliphatic C, condensates, and other elements such as N, H, and O—and environmental N sources. Interactions between PyOM and environmental N may influence gaseous N emissions, N leaching, N availability to living organisms, and global N transport^3^. Here, we focus on PyOM’s interactions with NH_3_—the atmosphere’s most abundant alkaline gas. Global NH_3_ emissions are projected to double by 2050 and constitute a large part of the Earth’s reactive N pool^4–6^. Common sources of NH_3_ in soils include decomposing organic matter, rainwater, and N fertilizer. Laboratory studies show that various forms of natural and industrially modified organic matter can retain NH_3_^7–18^, but the NH_3_ retention capacity of natural PyOM stocks and the mechanisms responsible for NH_3_ retention under ambient conditions have not been established. Therefore, the extent to which these studies can inform our understanding of PyOM’s role in global biogeochemical cycles is unknown.

Proposed mechanisms for NH_3_ retention by natural PyOM include physisorption, electrostatic interactions, and precipitation of ammonium (NH_4_^+^) salts^7–9^. Although these retention mechanisms would allow PyOM to act as a temporary N sink, N retained in these ways would be readily available for plant and microbial uptake, or loss through gas or solute transport^7^. Conversely, the formation of stronger covalent bonds between PyOM and NH_3_ would result in more persistent N retention, allowing PyOM to serve as a dynamic, long-term N source and sink—both capturing NH_3_ from its surroundings and slowly releasing it over time. This could also result in greater coupling of global C and N cycling, as covalently bound NH_3_–N would be carried with the PyOM–C as it traveled over great distances^1^. However, until now, covalent bond formation between natural PyOM and NH_3_ under ambient terrestrial conditions has not been observed.

Some evidence exists that under certain laboratory conditions, covalent bonds can form between NH_3_ and industrially produced relatives of PyOM or secondary organic aerosols found in the atmosphere. Graphene oxides and activated carbons can form a variety of cyclic and non-cyclic N structures when exposed to NH_3_ at temperatures exceeding 200 °C^10–14,17,18^. However, these materials are often modified (e.g., through exposure to chemical oxidants and heat or impregnated with metals), differ considerably from natural PyOM in surface area and functional group composition, and are exposed to NH_3_ under conditions that are not representative of the natural environment^19–21^. Thus, it is unknown whether these studies of graphene oxides and activated carbons can be used to predict interactions between natural PyOM and NH_3_, and whether the same variety of covalent N structures would develop under natural environmental conditions. Following exposure to NH_3_ at ambient temperatures, industrial relatives of PyOM can form non-cyclic amine and amide bonds^22^. Secondary organic aerosols found in the atmosphere—which contain functional groups present in terrestrial PyOM—can form both non-cyclic N structures as well as N heterocycles following exposure to NH_3_, NH_4_^+^, and amino acids under atmospheric conditions^23,24^. However, the formation of aromatic and non-aromatic heterocyclic N structures between terrestrial PyOM and NH_3_ has never been observed under ambient environmental conditions. This is of great interest because enrichment with these heterocyclic N structures influences the electrochemical properties, absorptive capacity, and environmental persistence of both natural and industrial pyrogenic C materials^16,22–27^. If heterocyclic N structures develop between PyOM and NH_3_ under natural conditions, this interaction would have important consequences for global C and N cycling.

In order to assess the impact of PyOM stocks on global nutrient cycles, it is also necessary to consider PyOM’s dynamic nature. Similar to the variety found in other sources of organic matter, different types of PyOM have different physical and chemical characteristics, including elemental makeup, functional group composition, surface area, pH, and other properties^28^. Additionally, PyOM properties change over time, as the material is exposed to water, sunlight, microbial activity, and other oxidizing forces^29–31^. Such variation in physiochemical properties can drastically alter PyOM’s role in the environment. Thus, to understand the influence of PyOM–NH_3_ interactions on global N cycling, it is important to consider how PyOM’s NH_3_ retention capacity might change over time. In this study, we investigate PyOM’s NH_3_ retention capacity under ambient conditions, N retention mechanisms, and whether retention capacity develops as PyOM stocks are weathered. We find that PyOM retains a surprising quantity of NH_3_–N and that this retention capacity increases significantly as PyOM is exposed to conditions mimicking natural weathering processes. More than half of the NH_3_–N is retained through chemisorption, including the formation of a variety of covalent bonds. We estimate that through these mechanisms soil PyOM stocks could play an important role in the global N cycle.

## Results

### Weathering increases PyOM N retention capacity

**Fig. 1:**
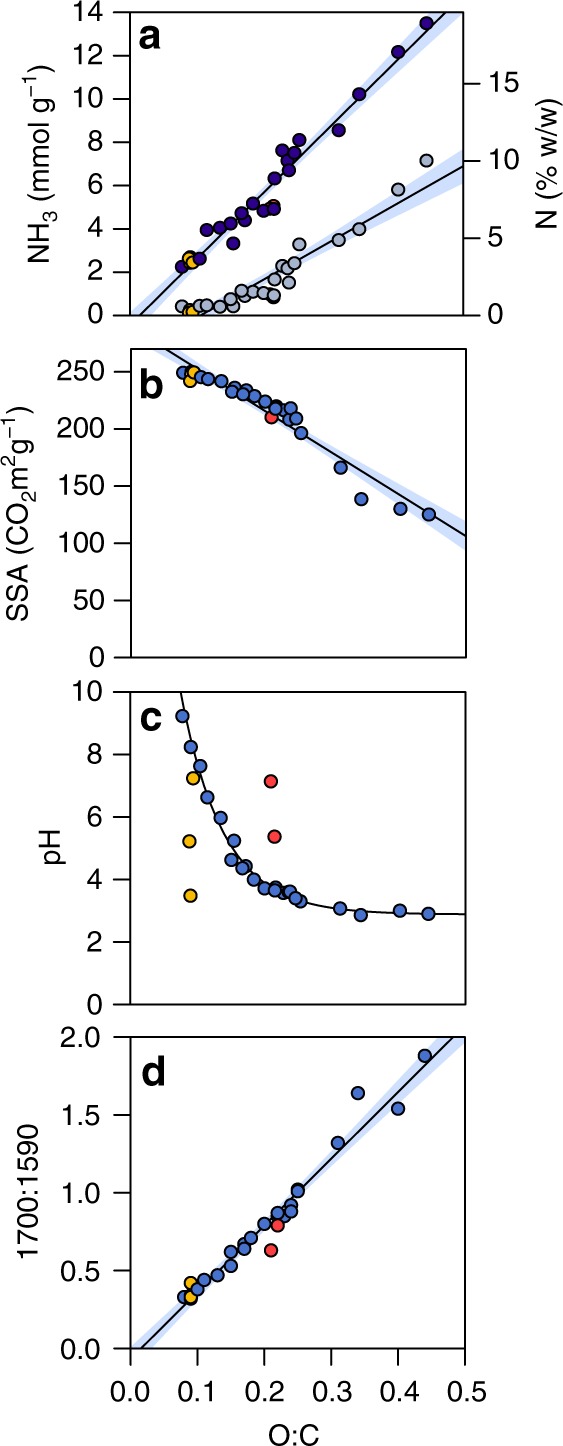
Changes in pyrogenic organic matter ammonia retention and physiochemical characteristics as a function of molar O:C ratio. **a** ammonia (NH_3_) retention capacity—expressed in mmol of NH_3_ g^−1^ of pyrogenic organic matter-carbon (PyOM–C, left *y* axis) and percent nitrogen (N) of PyOM–C (right *y* axis)—increases as a function of molar O:C ratio. Each point represents the average oxygen:carbon (O:C) ratio for two replicates. NH_3_ chemisorption = 17.49*x* − 1.84, *R*^2^ = 0.89, *p* < 0.001, F_1,25_ = 204.7, S_25_ = 0.59 (light blue); NH_3_ combined chemical and physical adsorption = 30.51*x* − 0.44, *R*^2^ = 0.96, *p* < 0.001, F_1,25_ = 567.2, S_25_ = 0.62 (dark blue). **b** Specific surface area (SSA) decreases as PyOM O:C ratio increases. SSA = −365*x* + 288.8, *R*^2^ = 0.931, *p* < 0.001, F_1,25_ = 338.5, S_25_ = 9.591. **c** PyOM pH decreases as oxidation increases. Blue symbols represent unoxidized PyOM and PyOM incubated with deionized water (DIH_2_O) and hydrogen peroxide (H_2_O_2_) and are fitted with a significant curve (*y* = 20.8*e^−14.8(O:C)^ + 2.84, S_19_ = 0.199). **d** The intensity of Fourier transform infrared (FTIR) peak heights associated with C=O stretching (1691–1715 cm^−1^) increases in proportion to the intensity of peak heights associated with C=C vibrations and stretching (1582–1609 cm^−1^) as PyOM O:C ratio increases (*y* = 4.29*x* − 0.0670, *R*^2^ = 0.963, *p* < 0.001, F_1,25_ = 650, S_25_ = 0.081). For all figures, yellow symbols represent PyOM that was incubated with 1 M hydrochloric acid (HCl); pink symbols represent PyOM that was incubated with H_2_O_2_ and then with 1 M sodium hydroxide (NaOH); shaded bands represent the 95% confidence intervals

PyOM produced from woody biomass was oxidized to generate a gradient of weathered PyOM^30,31^ and subsequently exposed to NH_3_ vapor at ambient temperature and pressure (35 °C and 80–800 Torr). Total NH_3_ capture increased more than sixfold after oxidation, from 2.3 mmol g^−1^ PyOM–C in unoxidized PyOM to 13.5 mmol g^−1^ PyOM–C in highly oxidized PyOM (Fig. 1a), showing that PyOM can retain substantial quantities of N from this form of NH_3_. Although specific surface area (SSA) and low pH may contribute to the NH_3_ retention capacity of some pyrogenic C materials^7,8,32^, these characteristics did not explain the trends observed here. PyOM SSA decreased with oxidation and therefore could not have contributed to the increase in NH_3_ retention observed in highly oxidized PyOM samples (Fig. 1b). PyOM pH also decreased with oxidation (Fig. 1c). However, when unoxidized PyOM was incubated with hydrochloric acid, which lowered its pH without altering key oxygen-containing functional groups (Supplementary Fig. 1), NH_3_ retention remained unchanged (Supplementary Fig. 2). This shows that although oxidation and low pH are correlated, pH itself did not drive NH_3_ retention and cannot be used to predict PyOM oxidation or NH_3_ retention capacity. Instead, our analyses indicate that functional group composition may be a more reliable determinant of PyOM’s NH_3_ retention capacity^11,21^. Peak height ratios measured by Fourier transform infrared spectroscopy (FTIR) and integrated peak areas measured by solid-state ^13^C nuclear magnetic resonance (NMR) spectroscopy suggest that with progressive oxidation, PyOM’s oxygen-containing functional groups increase relative to aromatic C structures (Fig. 1d and Supplementary Figs. 3 and 4). Quantitative stoichiometric measurements show that an increase in PyOM O:C ratio corresponds with the same trends observed through these spectral analyses. Taken together, these results highlight PyOM’s substantial and dynamic N retention capacity, and the relevance of weathering and exposure to oxidizing agents (e.g., microbial activity or ozone) when considering PyOM’s potential role in N cycling.

### PyOM retains NH_3_–N through chemisorption

**Fig. 2:**
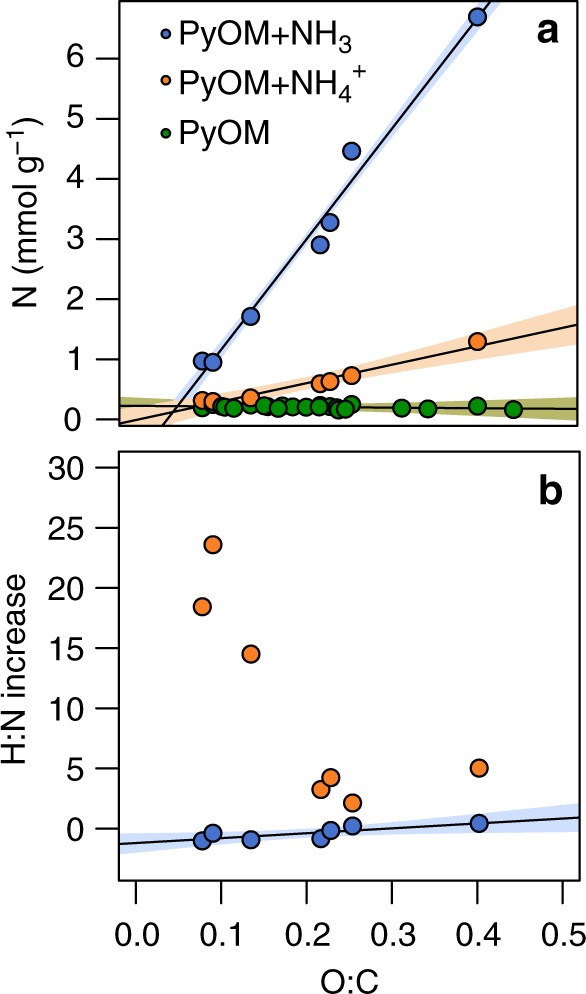
Pyrogenic organic matter N content and H:N ratio following exposure to ammonium or ammonia. **a** Pyrogenic organic matter (PyOM) nitrogen (N) retention in mmol g^−1^ PyOM–carbon (C) measured by dry combustion is significantly associated with molar O:C ratios following exposure to NH_3_ and NH_4_^+^ (*y* = 18.25*x* – 0.0665, *p* < 0.001 and *y* = 3.045*x* − 0.008, *p* < 0.001, respectively). **b** H:N molar increases are significantly associated with O:C ratios following exposure to NH_3_ (*y* = 6.23*x* − 1.70, *R*^2^ = 0.59, *p* < 0.005, F_1,10_ = 14.45, RSE_10_ = 0.53), but not NH_4_^+^. PyOM samples retain <0.5 moles of NH_3_–H for every mole of NH_3_–N retained, compared to 2.13–23.60 moles of NH_4_^+^–H for every mole of NH_4_^+^–N retained. Green symbols represent original PyOM samples without N addition, orange symbols represent PyOM following exposure to NH_4_^+^, and blue symbols represent PyOM following exposure to NH_3_. Shaded bands represent the 95% confidence intervals

In addition to revealing PyOM’s considerable NH_3_ retention capacity, adsorption isotherms showed that up to 53% of the NH_3_ was retained through chemisorption rather than physisorption, and that this proportion was greatest in oxidized PyOM (Fig. 1a). A commonly proposed mechanism for NH_3_ chemisorption by PyOM is protonation of NH_3_ to form NH_4_^+^ and subsequent electrostatic interaction between the NH_4_^+^ and PyOM’s negatively-charged functional groups^7^, but our data indicate that this mechanism cannot solely be responsible for PyOM NH_3_–N retention. Direct exposure of oxidized PyOM to NH_4_^+^ resulted in much lower N retention than exposure to NH_3_ gas (Fig. 2a), suggesting that electrostatic interactions alone cannot explain PyOM’s NH_3_ retention capacity. Furthermore, if physisorption or electrostatic interactions are predominantly responsible for PyOM–N retention from NH_3_ and NH_4_^+^, then stoichiometry dictates that on a molar basis, increases in PyOM–N following exposure should be accompanied by a threefold to fourfold increase in PyOM–H. However, when oxidized PyOM (molar O:C ratio 0.402) was exposed to NH_3_, the increase in PyOM molar H:N ratio was smaller than 0.5, suggesting that a substantial portion of NH_3_–N is retained without retention of NH_3_–H (Fig. 2b). When the same PyOM sample was exposed to NH_4_^+^, the molar H:N ratio increased by 5.03, suggesting that most of the NH_4_^+^–N was retained along with NH_4_^+^’s H atoms. This stoichiometric comparison of H:N ratios in PyOM samples before and after exposure to NH_3_ and NH_4_^+^ indicates that the respective mechanisms for N retention differ substantially, and that alternatives to physisorption and electrostatic interaction are likely responsible for PyOM’s NH_3_ retention capacity.

### PyOM and NH_3_–N form covalent bonds

**Fig. 3:**
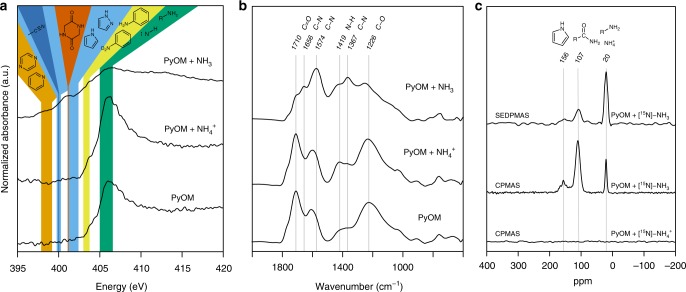
Nitrogen *K*-edge NEXAFS, FTIR, and NMR spectra of oxidized PyOM samples. Nitrogen (N) *K*-edge near-edge X-ray absorption fine structure (NEXAFS) (**a**), Fourier transform infrared (FTIR) (**b**), and nuclear magnetic resonance (NMR) (**c**) spectra collected from oxidized PyOM, oxidized PyOM following exposure to ammonium (NH_4_^+^), and oxidized pyrogenic organic matter (PyOM) following exposure to ammonia (NH_3_). **a** Shaded bands represent the range of peak centers consistent with selected spectral features: 397.88–399.20 eV for C=N bonds in 1N and 2N aromatic six-membered rings (orange), 400.00 for nitrile bonds (dark blue), 399.76–400.27 for C=N bonds in 2N five-membered rings (light blue), 401.15 for C–N bonds in non-aromatic six-membered rings (red), 401.20–402.40 for C–N bonds in 1N and 2N aromatic five-membered rings (light blue), 403.00–403.75 for aliphatic N bonded to aromatic rings (yellow), and 405.00–406.58 for aliphatic amines and N–H bonds (green). Model chemical structures are shown at the top of the figure. **b** FTIR spectra of oxidized PyOM, oxidized PyOM following exposure to NH_4_^+^, and oxidized PyOM following exposure to NH_3_. **c** ^15^N-NMR spin echo direct polarization magic angle spinning (SEDPMAS) spectrum of oxidized PyOM following exposure to [^15^N]-NH_3_, and ^15^N-NMR cross-polarization magic angle spinning (CPMAS) spectra of oxidized PyOM following exposure to [^15^N]-NH_3_ and [^15^N]-NH_4_^+^. The spectra suggest that NH_3_–N retention mechanisms could include NH_4_^+^ adsorption (represented by the peak at 20 ppm), and the formation of covalent C–N bonds such as amines (20 ppm), amides (~107 ppm), and aromatic five-membered heterocycles (chemical shifts between ~130–165 ppm)^16,33,39,40^

To examine these alternative mechanisms for NH_3_ retention, we compared the N near-edge X-ray absorption fine structure (*K*-edge NEXAFS) spectra of oxidized PyOM (O:C ratio 0.402) to those of oxidized PyOM following exposure to either NH_3_ or NH_4_^+^ (Fig. 3a and Supplementary Fig. 5). This method cannot be used to quantify the absolute amount of N retained by PyOM, but does provide information about the types of covalent N bonds present. Exposure to NH_3_ resulted in the formation of a variety of covalent N bonds that differ from those originating from PyOM feedstock N, N structures formed during thermal decomposition, N structures formed between PyOM and NH_4_^+^, and N–H bonds in pure NH_3_ or NH_4_^+^^33–36^. Interactions between oxidized PyOM and NH_3_ led to the strong development of absorption peaks between 397.88 and 402.40 eV, many of which are consistent with aromatic and non-aromatic heterocyclic N structures^14,36^ (see Supplementary Tables 1–3). Compared to spectra collected from PyOM that was not exposed to additional N, spectra collected from PyOM following exposure to NH_3_ showed a threefold increase in the 1*s* → π^*^ area consistent with aromatic six-membered heterocycles containing either one or two N atoms (model peaks located at 397.88, 398.76, and 399.2 eV), a threefold increase in the area consistent with nitrile bonds and aromatic five-membered heterocycles containing either one or two N atoms (400.05, 401.43, and 402.40 eV), and a 1.3-fold increase in the area consistent with aliphatic N bonded to aromatic rings (403.00 and 403.65 eV) (Supplementary Table 2). Additionally, while no feature consistent with non-aromatic six-membered N heterocycles (401.15 eV) was identified in spectra collected from oxidized PyOM not exposed to NH_3_, this feature accounted for two percent of the area underneath the spectrum collected from oxidized PyOM following exposure to NH_3_. In contrast, development of peaks in the 1*s* → π* region following exposure to NH_4_^+^ was very small, suggesting that there was little change in N functional group composition with NH_4_^+^ addition. Both the spectra of PyOM exposed to NH_4_^+^ as well as those of unexposed PyOM are strongly dominated by 1*s* → σ* features with peak centers between 405.00 and 406.58 eV, which dwarf the 1*s* → π* region area in these spectra. Although these 1*s* → σ* features are often associated with N–H bonds, they cannot be assigned definitively due to severe overlap of spectral features in this region^36^.

To further compare mechanisms for PyOM retention of NH_3_ in comparison to NH_4_^+^, we also collected FTIR spectra from oxidized PyOM, and oxidized PyOM following exposure to NH_3_ and NH_4_^+^ (Fig. 3b). Similar to the NEXAFS spectra (Fig. 3a), the FTIR spectra show clear differences between functional groups present in PyOM, PyOM following exposure to NH_3_, and PyOM following exposure to NH_4_^+^. Exposure to NH_3_ resulted in the emergence of new peaks at 1656 and 1367 cm^−1^ and an increase in peak height at 1574 cm^−1^, all of which are consistent with C–N stretching, including C–N resonance stretching in aromatic rings at 1656 cm^−1^^37,38^. Exposure to NH_3_ also resulted in a marked decrease in the peaks associated with C=O and C–O carbonyl/carboxyl and ketonic stretching at 1710 and 1226 cm^−1^, respectively, suggesting that these functional groups decrease relative to other functional groups in this sample. In contrast, exposure to NH_4_^+^ resulted in only two new spectral features, medium-sized peaks at 1419 and 1372 cm^−1^, which are consistent with N–H and C–N stretching, respectively.

Definitive functional group assignment using FTIR spectra collected from heterogeneous materials such as PyOM is challenging because the regions associated with different bonds often overlap with one another. However, the major treatment difference between our PyOM samples is whether or not they were exposed to gaseous NH_3_ or aqueous NH_4_^+^. Therefore, although FTIR spectral features between 1200 and 1700 cm^−1^ are sometimes associated with bonds between other elements (including C and O), it is probable that the emergence of distinct features in the FTIR spectra collected from our oxidized PyOM samples following exposure to either NH_3_ or NH_4_^+^ is a result of bonds that formed between PyOM and N. Since the FTIR spectrum of pure NH_3_ contains predominant peaks around 950 cm^−1^, it is also unlikely that physical adsorption of NH_3_ alone could account for the differences observed between the FTIR spectra collected from our PyOM samples before and after NH_3_ exposure^38^. In contrast, the FTIR spectra of NH_4_^+^ standards contain predominant peaks around 1440 cm^−1^, indicating that NH_4_^+^ adsorption was responsible for the relative increase in this region of the spectra collected from our PyOM samples following exposure to NH_4_^+^. This is consistent with our NEXAFS deconvolution analysis, which shows that exposure to NH_4_^+^ does not result in the substantial formation of a variety of N functional groups, despite the increased N content relative to unexposed PyOM samples. It also is consistent with elemental analyses, which suggest that oxidized PyOM retains NH_4_^+^–N in stoichiometric balance with NH_4_^+^–H, but retains NH_3_–N without NH_3_–H.

To further investigate mechanisms for NH_3_–N and NH_4_^+^–N retention, we collected solid-state ^15^N-NMR spectra after separate exposure of oxidized PyOM to enriched [^15^N]-NH_3_ gas or [^15^N]-NH_4_^+^ solution (Fig. 3c). Use of ^15^N-enriched reagents was necessary because there was insufficient signal from ^15^N at natural abundance in PyOM samples exposed to unlabeled NH_3_ or NH_4_^+^. Substantial differences were observed in the ^15^N-NMR CPMAS spectra collected from PyOM exposed to enriched [^15^N]-NH_3_ gas and these differences confirmed the formation of a variety of new N functional groups, including NH_4_^+^ and amines (~20 ppm), and C–N groups such as amides (~107 ppm) and N heterocycles (~156 ppm)^16,33,39,40^. Similar to the results of our NEXAFS spectral deconvolution analyses, integration of the ^15^N-NMR SEDPMAS spectrum collected from oxidized PyOM following exposure to [^15^N]-NH_3_ gas shows that over 40% of the newly-incorporated NMR-detectable N is consistent with covalent C–N bonds, including more than 11% in heterocyclic structures (Supplementary Table 4). On the other hand, the ^15^N-NMR spectrum collected from PyOM exposed to [^15^N]-NH_4_^+^ did not show any evidence of NH_4_^+^–N incorporation into PyOM, also corresponding with the results of our NEXAFS spectral deconvolution analysis, which show very little difference between the N functional group composition of PyOM and PyOM following exposure to NH_4_^+^. It is possible that some NH_4_^+^–N was incorporated into PyOM, but that the quantity retained was below the detection limit for NMR, implying that it is essential for an acid-base reaction (e.g., –CO_2_H + NH_3_ → –CO_2_–NH_4_^+^) to occur for NH_4_^+^ retention.

Direct comparison of NEXAFS and NMR results is difficult because of fundamental differences between the two methods. In particular, NMR detects functional groups, while NEXAFS detects individual bonds, some of which may be present together in one functional group. Additionally, due to severe overlap of features in the 1*s* → σ* region of NEXAFS N spectra, the portion of N–H and C–NH_2_ bonds present in a sample cannot be determined with this method. However, the overall results from the two analyses are consistent. In combination with stoichiometric analyses, the NEXAFS, NMR, and FTIR spectra show that PyOM interactions with NH_3_ under ambient conditions can result in substantial N retention and are fundamentally different from interactions with NH_4_^+^. The mechanism may involve nucleophilic NH_3_ reacting with predisposed functional groups of PyOM, such as acid anhydrides, or diketo-fragments to form a range of covalent C–N bonds, including amides and N heterocycles (Fig. 3). Similar reactions have been described between NH_3_/NH_4_^+^ and small organic molecules such as carbonyls, glyoxals, and secondary organic aerosols found in the atmosphere^23,24,41^. Both the NH_3_ retention capacity and mechanisms of natural PyOM are similar to those of some industrially produced graphene oxides and activated carbons^8,19,42^ even when exposure occurs at ambient temperature and pressure. These results demonstrate for the first time that the enrichment of PyOM with N functional groups such as N heterocycles, aromatic N heterocycles, and amides may occur under natural environmental conditions and that PyOM’s interaction with NH_3_ versus NH_4_^+^ has very different implications for the global N cycle.

## Discussion

Our data show that natural PyOM—a ubiquitous component of soil, atmosphere, and water—can react with NH_3_ gas to form covalent bonds under conditions approximating the natural environment. This is decisive because such covalent bond formation would result in more persistent N retention than physisorption, electrostatic interactions, and precipitation of NH_4_^+^ salts, which are currently thought to be the dominant mechanisms for PyOM NH_3_ retention^7–9^. Since covalently bound N might be less accessible to living organisms and less susceptible to volatilization, diffusion, and leaching than weakly sorbed NH_3_ and NH_4_^+^, it would also have very different implications for local N availability and global N cycling. By incorporating NH_3_–N into covalent C–N bonds, PyOM could provide a more dynamic mechanism for N storage, transport, and release. The discovery of aromatic and non-aromatic heterocyclic N bond formation between natural PyOM and NH_3_ at ambient temperatures is also noteworthy, as this has not been observed for terrestrial pyrogenic C material, including coal, activated carbon, and graphene oxides. This is particularly relevant for industrial applications, where N-doping is used to improve the performance of C-based supercapacitors, catalysts, and other materials^15,16,25^.

The formation of covalent bonds between PyOM and NH_3_–N under ambient conditions is a surprising outcome that has not been considered by most scientists investigating PyOM interactions with N. While the work presented here did not determine the chemical reactions responsible for such bond formation, similar reactions are well documented. For example, the reaction between carboxylic acids and amines (including NH_3_ as the simplest case) to form amides is the basis of protein synthesis from amino acids. It is also well established that subsequent condensation, cyclization, and aromatization to form N-aromatics are also possible. For example, the Paal–Knorr pyrrole synthesis reaction—which produces pyrroles through the condensation of a dicarbonyl compound with an amine or NH_3_—is thought to be responsible for N heterocycle formation between secondary organic aerosols and NH_3_ or amines in the atmosphere^23,24,41^. The discovery of covalent bond formation between PyOM and NH_3_ under ambient conditions may direct us to rethink PyOM material science and N biogeochemistry on local and global scales. Since many forms of organic matter present in the Earth’s soil, atmosphere, and water contain the same functional groups found in PyOM, it is possible that similar reactions occur between these materials and NH_3_. Future research should investigate the extent to which organic matter retains NH_3_–N through covalent bonds, the mechanisms responsible, and the implications for global N biogeochemistry.

Given the existing uncertainties in global PyOM and NH_3_ budgets, it is difficult to calculate exactly how much N might potentially be retained or transported by the Earth’s PyOM stocks. Based on estimates of 54–109 Pg PyOM–C in soil^1^ and an NH_3_ adsorption capacity of 13.5 mmol g^−1^ for oxidized PyOM (Fig. 1a), we calculate that soil PyOM stocks have the potential to store or transport up to 7.3–14.7 × 10^14^ mol NH_3_ through PyOM–NH_3_ interactions, equaling up to 645-fold more than estimated annual NH_3_ emissions from global agriculture, or up to 251-fold more than the estimated quantity of annually applied synthetic N fertilizer^43^. If NH_3_ interactions with soil PyOM are representative of those with other PyOM stocks, the atmospheric, ocean sediment, and marine PyOM pools could store or transport an additional 214 × 10^14^ mol NH_3_–N through similar mechanisms. Combined, all of these PyOM stocks could retain ~320 Pg N, or more than 1500-fold the contribution of global anthropogenic N inputs per year^44^.

These calculations predict a large potential influence of PyOM on global N cycling, and should motivate further work to constrain estimates so that they reflect the amount of NH_3_–N retained and transported by PyOM. It is important to consider factors influencing NH_3_ volatilization (e.g., pH, moisture, and temperature), PyOM–NH_3_ retention capacity (e.g., functional group composition, surface area, and fouling of PyOM surfaces), and other variables that affect interactions between PyOM and NH_3_ (e.g., the temperature of exposure, distance from the NH_3_ source, and biological competition for NH_3_). However, even at relatively low NH_3_ concentrations or PyOM–N retention levels, PyOM could influence NH_3_ loss, N availability to plants and microbes, and global N transport. Additional experiments are necessary to investigate the frequency of PyOM–NH_3_ interactions and to examine them in more complex and heterogeneous environments, especially in marine waters and sediments, which hold the vast majority of the Earth’s PyOM stocks. The coupling of global C and N cycles through such interactions could also be significant and warrants further research, particularly as global fire patterns change.

## Methods

### PyOM preparation

Maple (*Acer rubrum*) wood chips were pyrolyzed at 500 °C for 30 min in a modified muffle furnace^28^. In order to produce a homogenous product, the furnace employs a custom-made inline mixing unit, regulates temperature, and maintains an internal atmosphere of inert gas throughout pyrolysis. These highly standardized process conditions ensure that the pyrolysis products are as homogenous as possible. The resulting PyOM was ground and sieved to 149–850 µm, divided into subsamples, and incubated with hydrogen peroxide (H_2_O_2_) or deionized water (DIH_2_O) at 30 °C for up to three months (PyOM:H_2_O_2_ ratio of 1:10 g mL^−1^). After oxidation, PyOM was rinsed thoroughly with DIH_2_O and dried. Some PyOM samples were rewetted with DIH_2_O (PyOM: DIH_2_O ratio of 1:20 g mL^−1^) and treated with 1 M HCl or NaOH until the desired pH was achieved. These PyOM samples were also rinsed with DIH_2_O and dried.

### PyOM characterization

PyOM pH was measured in DIH_2_O at a ratio of 1:20 g mL^−1^. SSA was quantified using the B.E.T. method with CO_2_ at 273.15 K (ASAP 2020, Micromeritics, Atlanta, Georgia). Total C, N, H, and O were measured using a Delta V Isotope Ratio Mass Spectrometer (Thermo Scientific, Germany) coupled to a Carlo Erba NC2500 Elemental Analyzer (Italy).

### NH_3_ and NH_4_^+^ adsorption

NH_3_ adsorption isotherms were measured with an Autosorb iQ gas sorption analyzer (Quantachrome Instruments, Boynton Beach, Florida). Briefly, samples were degassed at 300 °C for 3 h prior to NH_3_ adsorption isotherm determination, which was conducted from 80 to 800 Torr at 35 °C. Chemisorption values indicate NH_3_ that was retained by PyOM under vacuum. See Supplementary Fig. 6 for comparison of N measured by chemisorption isotherms to N measured by dry combustion. For NH_4_^+^- adsorption measurements, PyOM samples were mixed with 100 mM ammonium chloride solution for 16 h, filtered, rinsed with ethanol, and dried. Retained N was measured using an elemental analyzer, as described above.

### FTIR

FTIR spectroscopy was used to characterize oxidized PyOM samples and investigate changes in PyOM functional group composition after exposure to NH_3_ and NH_4_^+^. Two replicates of each PyOM sample were scanned 200 times from 575 to 3500 cm^−1^ at a resolution of 8 cm^−1^ using a Bruker Hyperion FT-IR Spectrometer (Bruker, Billerica, Massachusetts) equipped with a ZnSe crystal source (PIKE Technologies, Inc., Madison, Wisconsin). Atmospheric background spectra were subtracted from each sample spectrum. Replicate sample spectra were averaged, baseline corrected, and normalized. Wavenumbers were assigned and peak ratios were calculated for the following functional groups: 752–761, 813–823, and 875–915 cm^−1^ to aromatic C–H out of plane deformation, 1690–1715 cm^−1^ to carbonyl/carboxyl and ketonic C=O stretching, and 1581–1609 cm^−1^ to aromatic C=C vibrations and stretching (OPUS, Bruker, Billerica, Massachusetts).

### NEXAFS

Nitrogen *K*-edge NEXAFS was used to discern how NH_3_ and NH_4_^+^ were retained by PyOM following exposure. Briefly, samples were mounted onto gold-coated silicon wafers and scanned in 49 different locations for 20 seconds each, without any spatial overlap to prevent radiation damage to the sample. N K_α_ partial fluorescence yield was collected using silicon drift detectors in the slew scanning mode of the spherical grating monochromator beamline at the Canadian Light Source (Saskatoon, Canada). For each sample, all 49 scans were averaged across four detectors and normalized by the beamline incident flux obtained by measuring the drain current in a gold mesh (IGOR Pro 6.36, WaveMetrics, Lake Oswego, Oregon). Following a modification of the method used by Gillespie et al.^45^, spectra were shifted based on the N_2_ absorption spectrum measured from ammonium sulfate, background corrected, smoothed, and normalized to an edge-step of 1 (Athena 0.8.056, Bruce Ravel; Ifeffit 1.2.11, Matt Newville, University of Chicago, Chicago, Illinois). Deconvolution was performed using Gaussian curves and peak characteristics of N-containing standards (Fityk 0.9.8, Marcin Wojdyr; see Supplementary Fig. 7 for N standard spectra, Supplementary Table 1 for peak assignments used in deconvolution, and Supplementary Table 3 for features in spectra collected from standard compounds). The fraction of π* area associated with specific N bonds (compared to total area of all deconvolution products) was calculated for each sample (Supplementary Table 2). If they were present as physically adsorbed molecules, neither NH_3_ nor NH_4_^+^ could have been responsible for the development of the numerous pre-edge features in the spectra collected from PyOM that was exposed to NH_3_ and NH_4_^+^ (see Supplementary Figs. 7 and 8, Supplementary Table 2, and refs. ^34–38^).

To confirm that radiation damage was not responsible for spectral features, samples were also scanned 15 additional times in the same location. These 15 spectra were then averaged, shifted, background corrected, smoothed, and normalized as described above. If the samples were susceptible to beam damage, we would expect to see new spectral features that would become more pronounced as each additional scan exposed the sample to increasing radiation. However, as shown in Supplementary Fig. 9, this did not occur—even after a 15-fold increase in radiation, the spectral features remain the same as those presented in Fig. 3a. As other authors have noted, this indicates that radiation damage was not responsible for the features present in NEXAFS sample spectra^46^.

### Solid-state NMR spectroscopy

Solid-state NMR spectroscopy was used to investigate how NH_3_ and NH_4_^+^ were retained by oxidized PyOM following exposure. In order to obtain a sufficiently strong signal during ^15^N-NMR experiments, oxidized PyOM samples were exposed to gaseous [^15^N]-NH_3_ with 98 atom% ^15^N (Air Liquide America Specialty Gases, Plumsteadville, PA) and [^15^N]-NH_4_^+^ with 10 atom% ^15^N (Cambridge Isotope Labs, Tewksbury, MA) at 35 °C, under atmospheric pressure.

1D ^1^H, ^13^C and ^15^N solid-state NMR spectra were obtained at a magnetic field of 7 Tesla (^1^H, ^13^C and ^15^N Larmor frequency of 300 MHz, 75 and 30 MHz, respectively) using a Bruker Avance III NMR spectrometer fitted with a 4 mm magic angle spinning (MAS) double resonance probe. For both the ^13^C and ^15^N experiments, ~50 mg of PyOM was packed into a 4 mm zirconia rotor sealed with a Kel-F cap. For ^13^C experiments, CP (cross-polarization) was achieved with 6.5 kHz MAS; contact time, 1 ms, ramped from 70 to 100%; recycle delay, 3–20 s (Supplementary Figs. 10 and 11); 83 kHz ^1^H decoupling via spinal-64 sequence; ca 2k scans; TOSS (TOtal Suppression of Spinning side-bands) removed side-bands from the aromatic peaks that obscured the aliphatic region, and also gave better quality response from the sample without pulse breakthrough (Supplementary Figs. 4 and 12); SEDP (spin echo direct polarization with ^1^H decoupling) was achieved with 12 kHz MAS; recycle delay of 2–150 s; ^13^C excitation pulse of 4.5 µs (90°); echo time of ~75 µs; 71 kHz ^1^H decoupling via spinal-64 sequence (Supplementary Fig. 13). Complementary spectra were also acquired with dipolar-dephasing delays of 40 µs with both CP and SEDP to assess non-protonated carbon content. For ^15^N experiments, CP was achieved at 5 kHz MAS; contact time, 2 ms, ramped from 70 to 100%; recycle delay, 3 s; 83 kHz ^1^H decoupling via spinal-64 sequence^47^. TOSS was also used to suppress pulse breakthrough. For ^15^N SEDPMAS experiments, 10 kHz MAS was used with 100 µs echo time and a 50 kHz ^1^H decoupling field during acquisition with the spinal-64 sequence. The relaxation behavior was tested using a series of experiments with a fixed number of scans (400) and with increasing recycle delays from 5 to 400 s (Supplementary Figs. 14 and 15).

All spectra were processed using the Bruker software, TOPSPIN 3.5pl7. Spectra were produced from the free induction decays by first zero filling, applying Gaussian multiplication (e.g., LB = −10, GB = 0.03), Fourier transformation, and phase correction. Chemical shift values were referenced to the C=O of glycine, δ_C_ 176 ppm for ^13^C and to (NH_4_)_2_SO_4_, δ_N_ 24 ppm on the NH_3_ scale for ^15^N. All literature quoted in the text were converted to the NH_3_ scale for ^15^N by adding 380 ppm.

### Data analysis

All statistical analyses were performed using the lsmeans and nlstools packages^48,49^ in the statistical computing language and environment R^50^.

## Supplementary information

Supplementary Information

Peer Review File

## Data Availability

The data that support the findings of this study are available in Cornell University’s digital repository eCommons with the identifier 10.7298/X0B7-PX55.

## References

[1] Bird MI, Wynn JG, Saiz G, Wurster CM, McBeath A (2015). The pyrogenic carbon cycle. Annu. Rev. Earth Planet. Sci..

[2] Lauer A, Hendricks J (2006). Simulating aerosol microphysics with the ECHAM4/MADE GCM—Part II: Results from a first multiannual simulation of the submicrometer aerosol. Atmos. Chem. Phys..

[3] Clough TJ, Condron LM, Kammann C, Müller C (2013). A review of biochar and soil nitrogen dynamics. Agron. J..

[4] Galloway JN (2004). Nitrogen cycles: past, present, and future. Biogeochemistry.

[5] Clarisse L, Clerbaux C, Dentener F, Hurtmans D, Coheur PF (2009). Global ammonia distribution derived from infrared satellite observations. Nat. Geosci..

[6] Krupa SV (2003). Effects of atmospheric ammonia (NH_3_) on terrestrial vegetation: a review. Environ. Pollut..

[7] Taghizadeh-Toosi A, Clough TJ, Sherlock RR, Condron LM (2012). Biochar adsorbed ammonia is bioavailable. Plant Soil.

[8] Kastner JR, Miller J, Das KC (2009). Pyrolysis conditions and ozone oxidation effects on ammonia adsorption in biomass generated chars. J. Hazard. Mater..

[9] Day D, Evans RJ, Lee JW, Reicosky D (2005). Economical CO_2_, SO_x_, and NO_x_ capture from fossil-fuel utilization with combined renewable hydrogen production and large-scale carbon sequestration. Energy.

[10] Zawadzki J, Wisniewski A (2003). In situ characterization of interaction of ammonia with carbon surface in oxygen atmosphere. Carbon N. Y..

[11] Stohr B, Boehm HP, Schlogl R (1991). Enhancement of the catalytic activity of activated carbons in oxidation reactions by thermal treatment with ammonia or hydrogen cyanide and observation of a superoxide species as a possible intermediate. Carbon N. Y..

[12] Jansen RJJ, Van Bekkum H (1994). Amination and ammoxidation of activated carbons. Carbon N. Y..

[13] Schultz BJ (2014). X-ray absorption spectroscopy studies of electronic structure recovery and nitrogen local structure upon thermal reduction of graphene oxide in an ammonia environment. Rsc Adv..

[14] Geng D (2011). Nitrogen doping effects on the structure of graphene. Appl. Surf. Sci..

[15] Li X (2009). Simultaneous nitrogen doping and reduction of graphene oxide. J. Am. Chem. Soc..

[16] Latham KG, Rawal A, Hook JM, Donne SW (2016). Molecular structures driving pseudo-capacitance in hydrothermal nanostructured carbons. Rsc Adv..

[17] Mortland MM (1958). Reactions of ammonia in soils. Adv. Agron..

[18] Richardson LB (1917). The adsorption of carbon dioxide and ammonia by charcoal. J. Am. Chem. Soc..

[19] Zhang TY (2004). Preparation of activated carbon from forest and agricultural residues through CO_2_ activation. Chem. Eng. J..

[20] Azargohar R, Dalai AK (2006). Biochar as a precursor of activated carbon. Appl. Biochem. Biotechnol..

[21] Seredych M, Bandosz TJ (2007). Mechanism of ammonia retention on graphite oxides: Role of surface chemistry and structure. J. Phys. Chem. C.

[22] Petit C, Seredych M, Bandosz TJ (2009). Revisiting the chemistry of graphite oxides and its effect on ammonia adsorption. J. Mater. Chem..

[23] Updyke KM, Nguyen TB, Nizkorodov SA (2012). Formation of brown carbon via reactions of ammonia with secondary organic aerosols from biogenic and anthropogenic precursors. Atmos. Environ..

[24] De Haan DO (2009). Secondary organic aerosol-forming reactions of glyoxal with amino acids. Environ. Sci. Technol..

[25] Li XF (2016). Unraveling the formation mechanism of graphitic nitrogen-doping in thermally treated graphene with ammonia. Sci. Rep..

[26] de la Rosa JM, Knicker H (2011). Bioavailability of N released from N-rich pyrogenic organic matter: an incubation study. Soil Biol. Biochem..

[27] Smernik RJ, Baldock JA (2005). Does solid-state ^15^N NMR spectroscopy detect all soil organic nitrogen?. Biogeochemistry.

[28] Enders A, Hanley K, Whitman T, Joseph S, Lehmann J (2012). Characterization of biochars to evaluate recalcitrance and agronomic performance. Bioresour. Technol..

[29] Velasco-Molina M, Berns AE, Macias F, Knicker H (2016). Biochemically altered charcoal residues as an important source of soil organic matter in subsoils of fire-affected subtropical regions. Geoderma.

[30] Cheng CH, Lehmann J, Thies JE, Burton SD, Engelhard MH (2006). Oxidation of black carbon by biotic and abiotic processes. Org. Geochem..

[31] Cheng CH, Lehmann J, Engelhard MH (2008). Natural oxidation of black carbon in soils: Changes in molecular form and surface charge along a climosequence. Geochim. Cosmochim. Acta.

[32] Park SJ, Jin SY (2005). Effect of ozone treatment on ammonia removal of activated carbons. J. Colloid Interf. Sci..

[33] Knicker H (2007). How does fire affect the nature and stability of soil organic nitrogen and carbon? A review. Biogeochemistry.

[34] Jaeger R, Stohr J, Kendelewicz T (1983). X-ray induced electron stimulated desorption versus photon stimulated desorption: NH_3_ on Ni(110). Surf. Sci..

[35] Wight GR, Brion CE (1974). K-shell excitation of CH_4_, NH_3_, H_2_O, CH_3_OH, CH_3_OCH_3_ and CH_3_NH_2_ by 2.5 eV electron impact. J. Electron Spectrosc..

[36] Leinweber P (2007). Nitrogen K-edge XANES—an overview of reference compounds used to identify ‘unknown’ organic nitrogen in environmental samples. J. Synchrotron Radiat..

[37] Gunasekaran S, Sailatha E, Seshadri S, Kumaresan S (2009). FTIR, FT Raman spectra and molecular structural confirmation of isoniazid. Indian J. Pure Appl. Phys..

[38] National Institute of Standards and Techology. NIST Chemistry WebBook. 10.18434/T4D303 (2016).

[39] Knicker H (2002). The feasibility of using DCPMAS ^15^N ^13^C NMR spectroscopy for a better characterization of immobilized ^15^N during incubation of ^13^C- and ^15^N-enriched plant material. Org. Geochem..

[40] Smernik RJ, Baldock JA (2005). Solid-state ^15^N NMR analysis of highly ^15^N-enriched plant materials. Plant Soil.

[41] Amarnath V (1991). Intermediates in the Paal–Knorr synthesis of pyrroles. J. Org. Chem..

[42] Guo X, Tak JK, Johnson RL (2009). Ammonia removal from air stream and biogas by a H_2_SO_4_ impregnated adsorbent originating from waste wood-shavings and biosolids. J. Hazard. Mater..

[43] Beusen AHW, Bouwman AF, Heuberger PSC, Van Drecht G, Van Der Hoek KW (2008). Bottom-up uncertainty estimates of global ammonia emissions from global agricultural production systems. Atmos. Environ..

[44] Fowler D (2013). The global nitrogen cycle in the twenty-first century. Philos. Trans. R. Soc. B.

[45] Gillespie AW (2014). Nitrogen input quality changes the biochemical composition of soil organic matter stabilized in the fine fraction: a long-term study. Biogeochemistry.

[46] Gillespie AW (2015). Advances in using soft X-ray spectroscopy for measurement of soil biogeochemical processes. Adv. Agron..

[47] Mao J, Cao X, Olk DC, Chu W, Smidt-Rohr K (2017). Advanced solid-state NMR spectroscopy of natural organic matter. Prog. Nucl. Mag. Reg. Spectrosc..

[48] Lenth R (2016). Least-squares means. The R package lsmeans. J. Stat. Softw..

[49] Baty F (2015). A toolbox for nonlinear regression in R: the package nlstools. J. Stat. Softw..

[50] R Development Core Team. *R: A Language and Environment for Statistical Computing*. http://www.R-project.org/ (2011).

