# Supplementary material for: Fire-derived organic matter retains ammonia through covalent bond formation
Source: Nat Commun. 2019 Feb 8;10:664. doi: 10.1038/s41467-019-08401-z (PMC6368596; doi:10.1038/s41467-019-08401-z)
Supplement: Supplementary file 1 — Supplementary Information [file 41467_2019_8401_MOESM1_ESM.pdf]

Supplementary Information for

Fire-derived organic matter retains ammonia through covalent bond formation

Rachel Hestrin[1], Dorisel Torres-Rojas[1], James J. Dynes[2], James M. Hook[3], Tom Z. Regier[2], Adam W. Gillespie[2,6], Ronald J. Smernik[4] and Johannes Lehmann[1,5,*]

[1]*Soil and Crop Sciences, School of Integrative Plant Science, Bradfield Hall, Cornell University, Ithaca, NY 14853, USA*

[2]*Canadian Light Source Inc., 44 Innovation Boulevard, Saskatoon, SK S7N 2V3, Canada*

[3]*NMR Facility & Spectroscopy Lab, Mark Wainwright Analytical Centre and School of Chemistry, University of New South Wales, Sydney, NSW 2052, Australia*

[4]*School of Agriculture, Food and Wine, The University of Adelaide, Waite Campus, Urrbrae, SA 5064, Australia*

[5]*Atkinson Center for a Sustainable Future, Rice Hall, Cornell University, Ithaca, NY 14853, USA*

[6] *Present Address: School of Environmental Sciences, University of Guelph, Guelph, ON, N1G 2W1, Canada*

**corresponding author: CL273@cornell.edu*

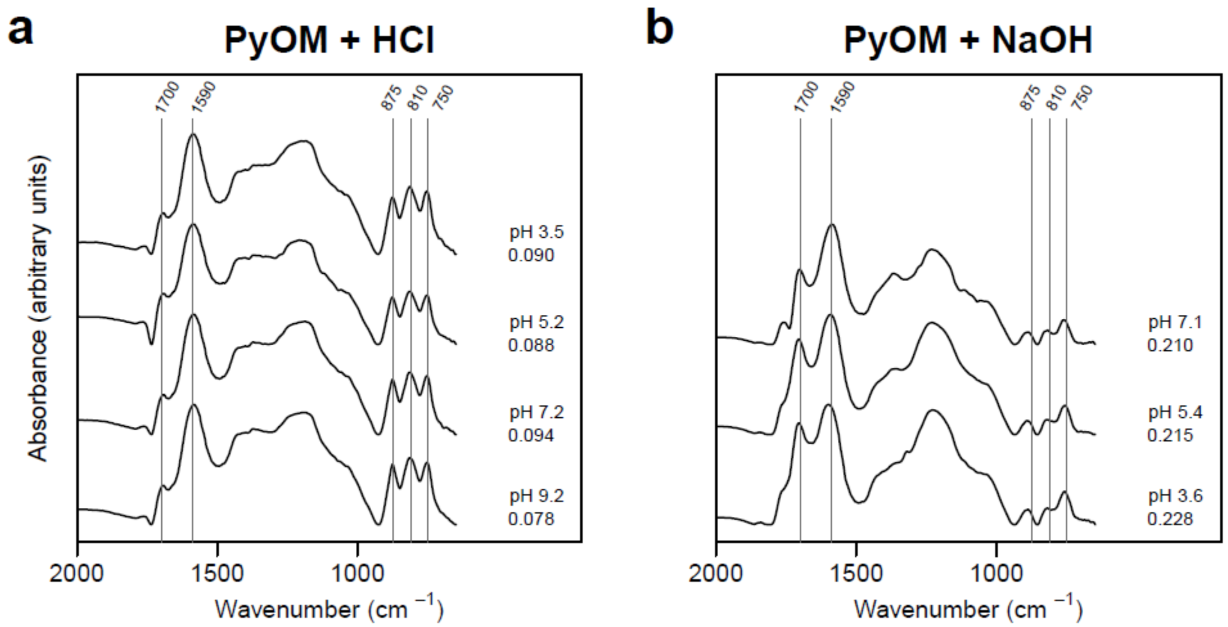

Supplementary Figure 1. FTIR spectra of PyOM samples after treatment with acid or base. (a) PyOM samples incubated with 1M HCl to achieve low pH without oxidation. (b) PyOM samples incubated with H_2O_2 and then with 1M NaOH to produce samples that have undergone oxidation, but have a high pH. Right-hand labels in both figures indicate molar O:C ratio below pH after incubation. FTIR spectral features suggest that incubation with acid does not influence functional group composition. In contrast, incubation with base results in a relative decrease in oxygen functional groups.

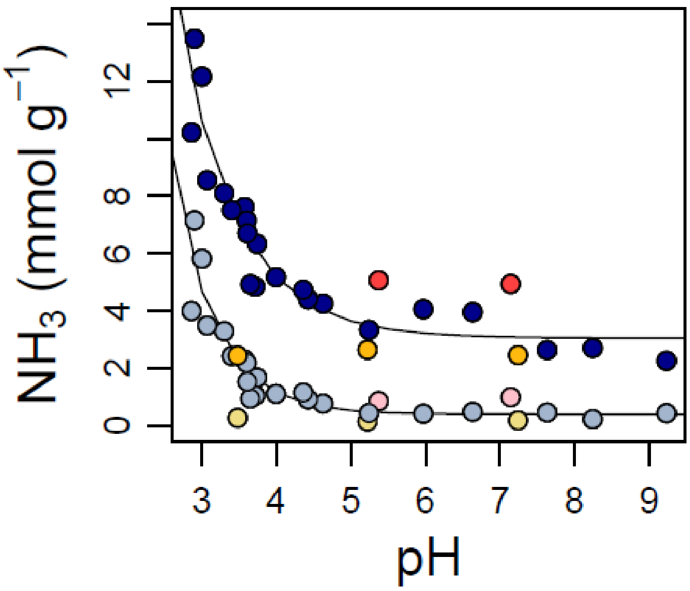

Supplementary Figure 2. Total NH_3 adsorption (dark blue, pink and yellow) and chemisorption (light blue, pink and yellow) to PyOM. Blue symbols represent unoxidized PyOM and PyOM incubated with DIH_2O and H_2O_2, and are fitted with significant curves (NH_3 Chemisorption: $y = 6.65*e^{-1.75*(pH-2.75)} + 0.39$, $S_{19} = 0.72$; NH_3 Combined Adsorption: $y = 10.40*e^{-1.27*(pH-2.75)} + 3.05$, $S_{19} = 0.997$). Yellow symbols represent PyOM samples that were incubated with 1M HCl; pink symbols represent PyOM samples that were incubated with 1M NaOH following incubation with H_2O_2.

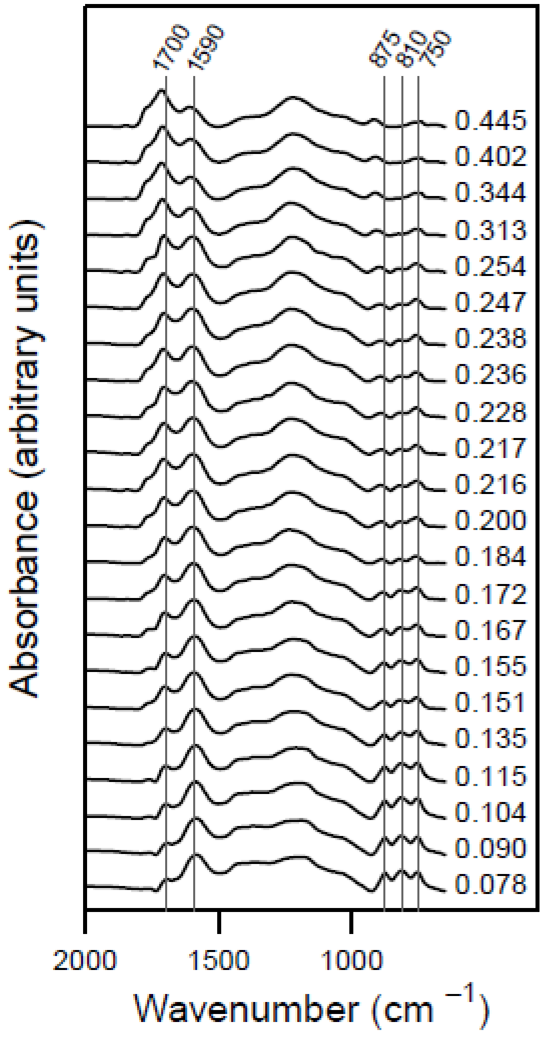

Supplementary Figure 3. FTIR spectra of PyOM samples along an oxidation gradient. Right-hand labels indicate molar O:C ratio. Lines (from left to right) represent carbonyl/carboxyl and ketonic C=O stretching at 1700 cm^{-1}, aromatic C=C vibrations and stretching at 1590 cm^{-1}, and aromatic C-H out of plane deformation at 875, 810, and 750 cm^{-1}. As oxidation progresses, there is an increase in the peak height ratio of the peaks associated with C=O bonds compared to peaks associated with C=C bonds, and an overall height decrease in peaks associated with C-H. This reveals a gradual shift in composition of PyOM functional groups, likely related to a decrease in aromatic structures and an increase in oxygen-containing functional groups.

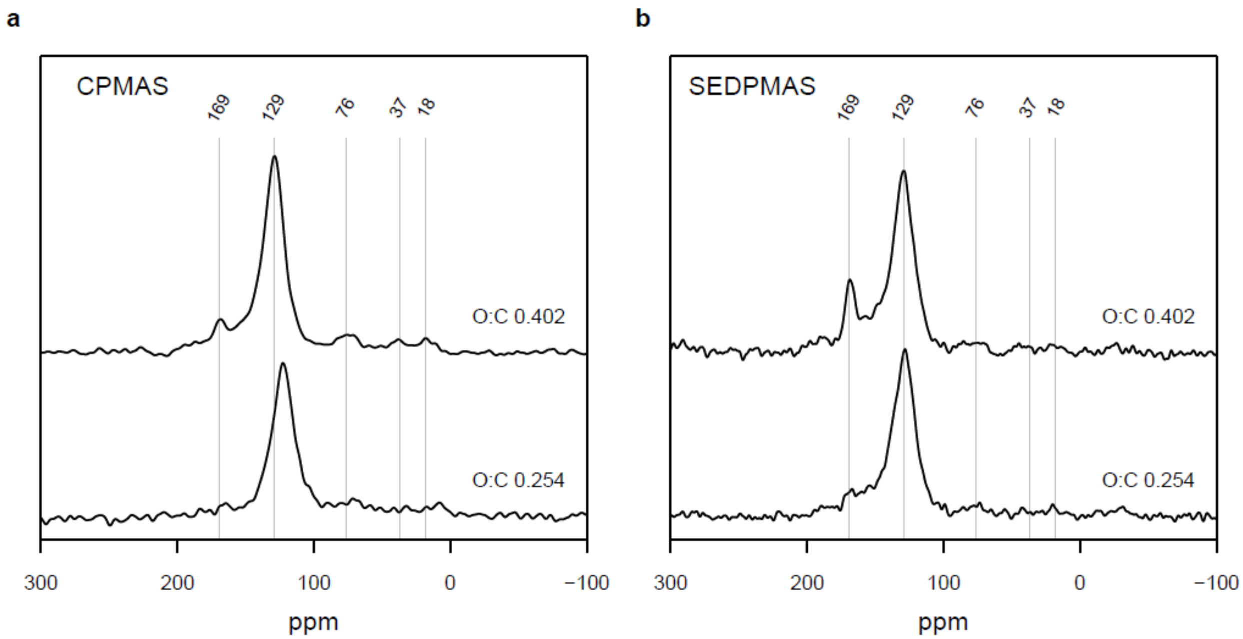

Supplementary Figure 4. Solid state ^{13}C CPMAS-TOSS (a) and SEDPMAS (b) NMR spectra collected from oxidized PyOM samples. PyOM molar O:C ratios are indicated in the right-hand corner next to each spectrum. Spectra show greater evidence of carboxyl groups in highly oxidized PyOM (chemical shift centered around 169 ppm) compared to less oxidized PyOM[1]. The chemical shift centered around 129 ppm is consistent with aromatic C; shifts between 18-76 ppm is consistent with aliphatic C[1].

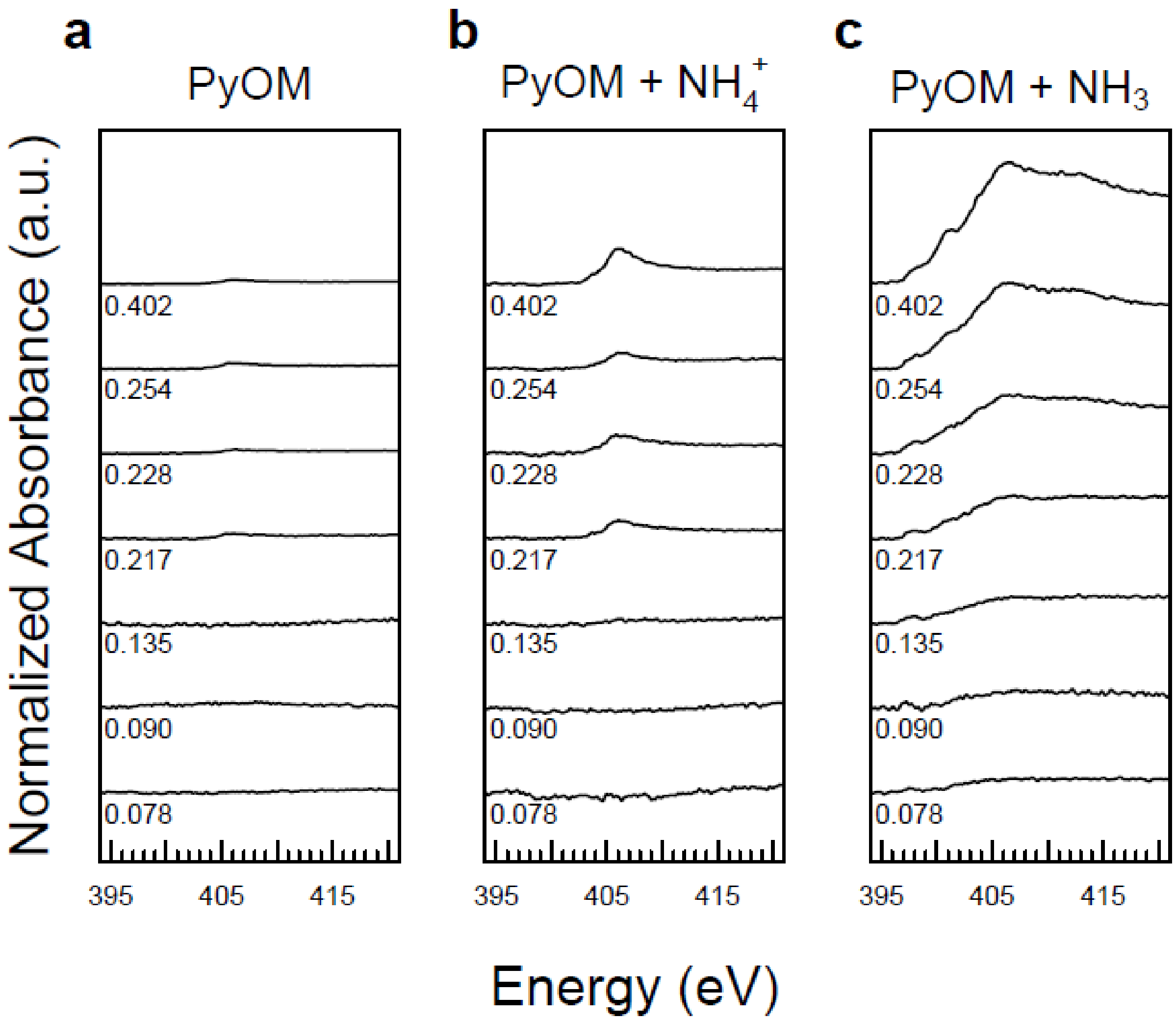

Supplementary Figure 5. Nitrogen *K*-edge NEXAFS spectra of PyOM samples normalized by N content. (a) PyOM, (b) PyOM exposed to NH_4^+, and (c) PyOM exposed to NH_3. Spectra are arranged from bottom to top according to degree of oxidation, indicated by the molar O:C ratio prior to exposure to additional N on the left side of each spectrum.

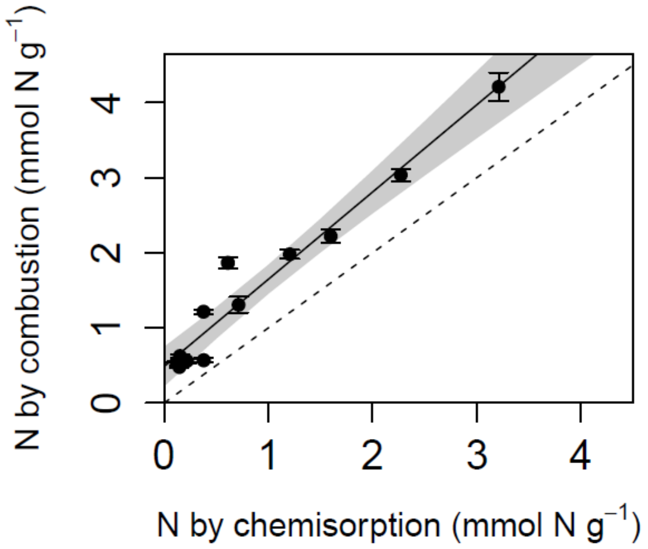

Supplementary Figure 6. N measured by NH_3 chemisorption compared to N measured by dry combustion. Black circles represent mean mmol N g^{-1} retained by the PyOM after exposure to NH_3, as measured by chemisorption (x axis) and dry combustion (y axis). Mean PyOM N content prior to NH_3 exposure was subtracted from dry combustion values in order to represent post-exposure N only. Error bars show the SE (n = 2). The data are fitted by a significant linear regression (N retained by PyOM as measured by dry combustion = 0.486 - 1.161*N retained by PyOM as measured by NH_3 adsorption isotherm; $p < 0.001$, $F_{1, 10} = 188.9$, $R^2 = .95$). The shaded area shows the upper and lower 95% CLs for the linear regression. The dashed line represents the 1:1 line, showing that NH_3 chemisorption isotherms predict lower N content than dry combustion.

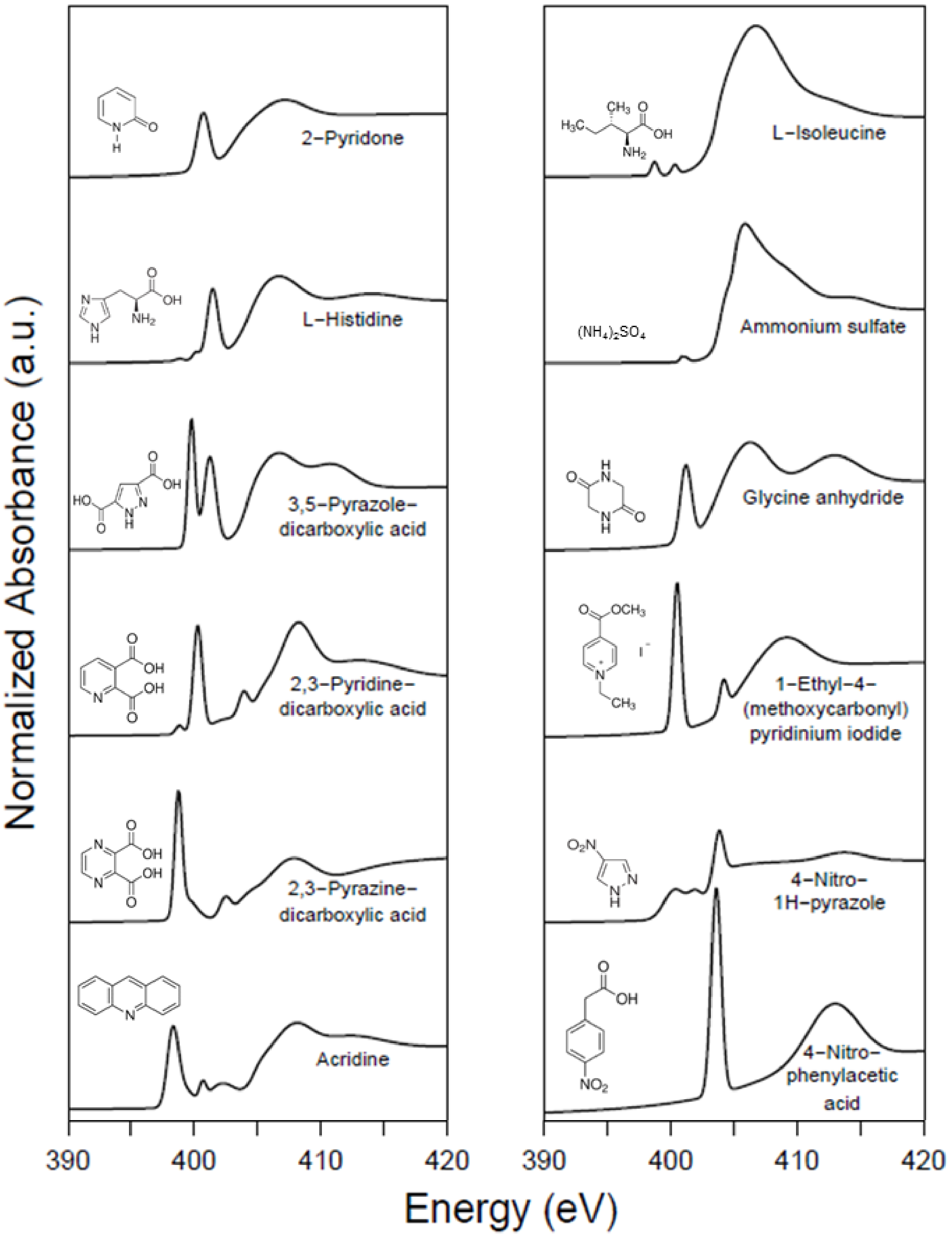

Supplementary Figure 7. N *K*-edge NEXAFS spectra of N standard compounds. See Supplementary Table 3 for specific peak values.

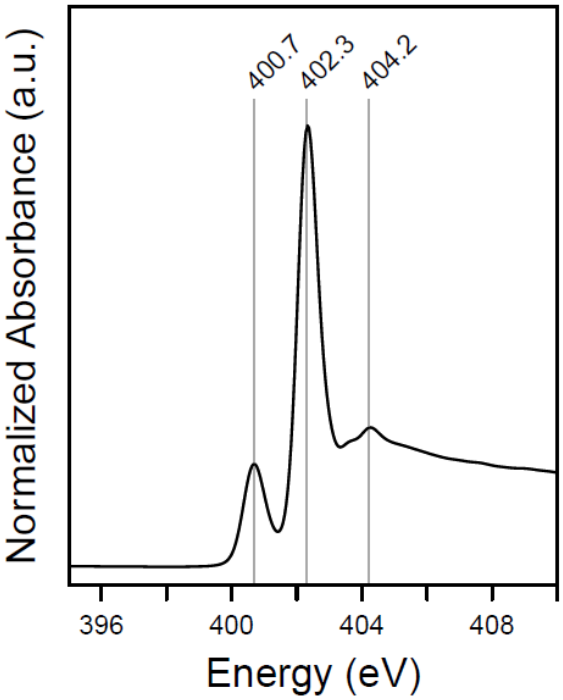

Supplementary Figure 8. N *K*-edge NEXAFS spectrum of NH_3 gas. We collected the partial fluorescence yield of NH_3 gas, in order to confirm that the features present in the spectrum of PyOM following exposure to NH_3 were not due to NH_3 gas itself. The NH_3 gas spectrum shows major features at 400.7, 402.3, and 404.2 eV, corresponding with NEXAFS and ISEELS NH_3 spectra collected by other authors[2-4]. The distinct NH_3 gas peaks cannot account for the broad features of the NEXAFS spectrum of PyOM that has been exposed to NH_3 (see Fig. 3a). In particular, the NH_3-exposed PyOM spectrum contains prominent features below 400 eV, which is the region most closely associated with aromatic 6-membered heterocyclic N structures and with nitrile bonds. Similarly, the peaks present in the FTIR spectra of NH_3 gas could not account for the differences between the FTIR spectra of oxidized PyOM and NH_3-exposed PyOM[5]. Taken together, these data provide further evidence that a variety of covalent N bonds form when NH_3 interacts with PyOM at ambient temperature.

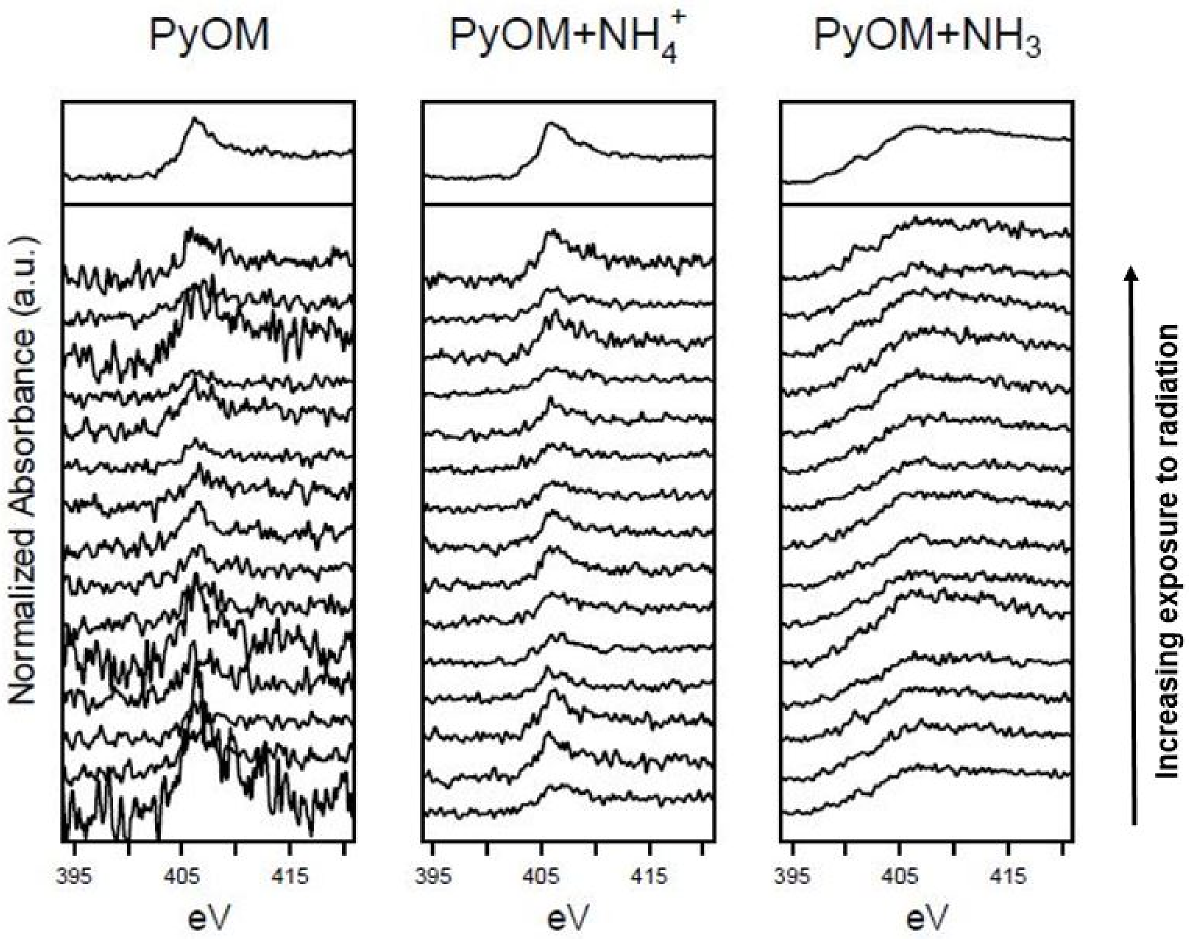

Supplementary Figure 9. Beam damage tests for N *K*-edge NEXAFS spectra. Fifteen scans were collected from the same location in each sample (PyOM, PyOM following exposure to NH_4^+, and PyOM following exposure to NH_3), thereby exposing each sample to fifteen-fold the dose of radiation that was used for other analyses (e.g., spectra presented in Fig. 3a and deconvolution results presented in Supplemental Table 2). Spectra above are arranged in the order that they were collected, so that the first scan collected is shown at the bottom of the figure and the following fifteen scans are shown in ascending order. An average of all fifteen scans is shown in the panel at the top of the figure. Repeated exposure to X-ray radiation did not create notable changes in spectral features, indicating that beam damage did not occur during collection of NEXAFS spectra. The averaged spectra in the top panel do not contain spectral features that

are distinct from the averaged spectra collected from different locations in each sample (Fig. 3a), further indicating that exposure to X-rays did not damage the samples or introduce artifacts into the NEXAFS spectra collected from these samples.

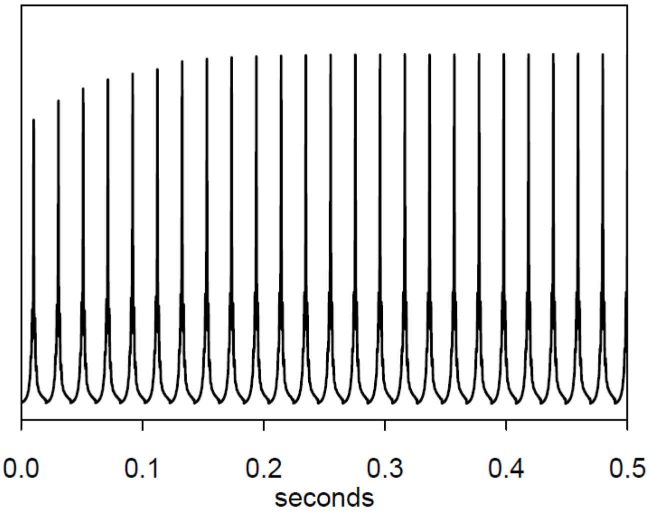

Supplementary Figure 10. ^{1}H relaxation of oxidized PyOM. This verifies that the T_1 relaxation of the bulk protons in the PyOM sample is less than 100 ms, which shows that a recycle delay of 3 s is more than adequate for ^{13}C CPMAS. The experiment is an array, varying D1 (the relaxation delay) in 20 ms increments, with ns = 4 and MAS = 10 kHz. Only the isotropic peak has been plotted in the array for clarity.

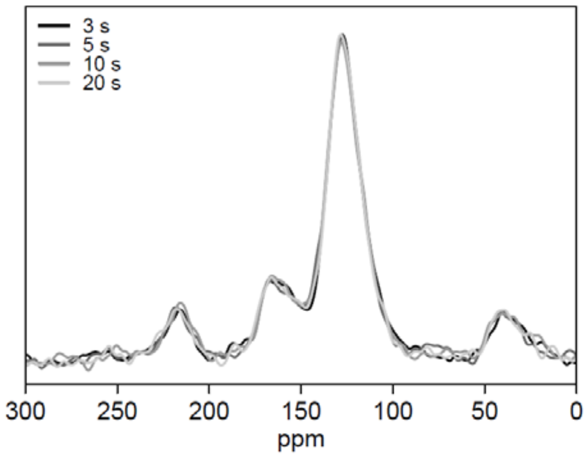

Supplementary Figure 11. ^{13}C CPMAS spectra collected from oxidized PyOM using a recycle delay time of 3 – 20 seconds; contact time: 1 ms; ns = ca 2,000; MAS = 6.5 kHz. This shows that a recycle delay of 3 s is adequate for ^{13}C CPMAS and that increasing the recycle delay time does not change or improve the spectra collected from the PyOM samples, as would be expected when the $T_1H < 100$ms. Spinning side bands are evident at ~220 and 40 ppm.

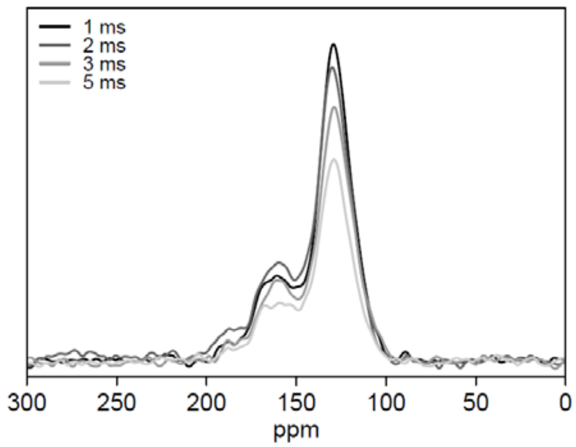

Supplementary Figure 12. ^{13}C CPMAS-TOSS spectra collected from oxidized PyOM using a contact time of 1 – 5 ms; relaxation delay, D1 = 3; ns = ca 2,000; MAS 6.5 kHz. This verifies that 1 ms is the optimum contact time for observing ^{13}C with CPMAS-TOSS spectra collected from the PyOM samples. The signal decreases when a longer contact time is used.

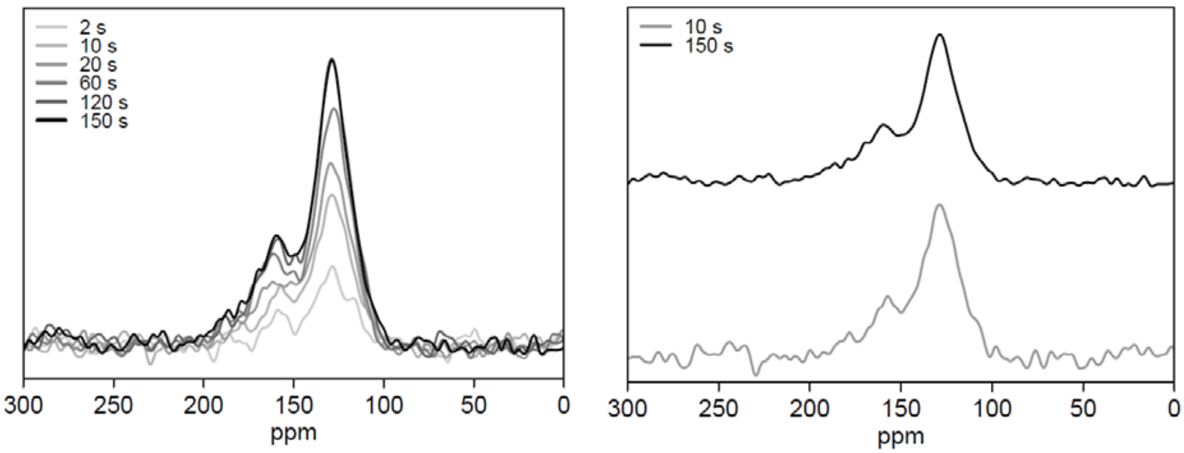

Supplementary Figure 13. ^{13}C SEDPMAS spectra collected from oxidized PyOM using a relaxation delay of 2 – 150 s; ns = 256; MAS = 12 kHz. Left pane: SEDPMAS relaxation delay experiment. Right pane: Normalized SEDPMAS spectra for peak ratio comparison of spectra collected using a relaxation delay of 10 vs 150 s. These spectra show that full relaxation occurs around ~120 s. However, the relative ratio of peaks within each spectrum is similar for spectra collected with a relaxation delay of 10 s and 150 s, suggesting that overall interpretation of the results will not change based on an increase in relaxation delay time.

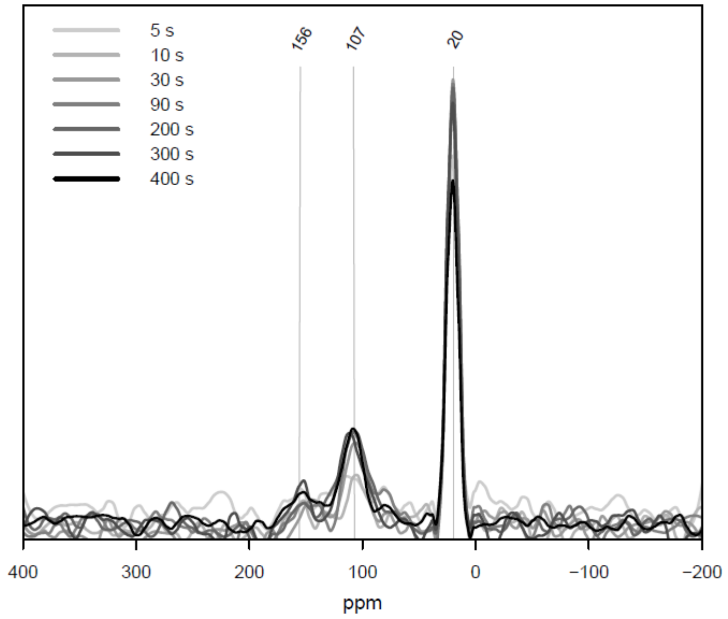

Supplementary Figure 14. ^{15}N SEDPMAS NMR spectra collected from oxidized PyOM after treatment with [^{15}N]-NH_3 using a relaxation delay of 5 – 400 s; ns = 400; MAS = 10 kHz. These spectra show that a relaxation delay of 300 s is sufficient to achieve full relaxation.

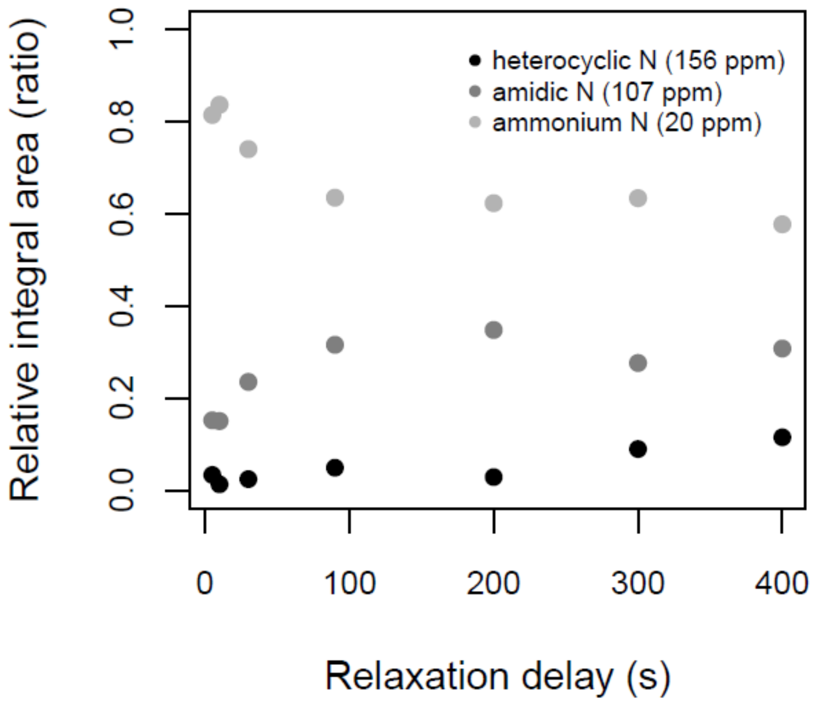

Supplementary Figure 15. Relative integral area of spectral features consistent with N functional groups represented in ^{15}N SEDPMAS NMR spectra collected from oxidized PyOM after treatment with [^{15}N]-NH_3 using a relaxation delay of 5 – 400 s. Trends in the ratio representing each N functional group indicate that a 300 s relaxation delay is sufficient to achieve full relaxation.

Supplementary Table 1. N *K*-edge NEXAFS peak assignments used in deconvolution model.

N Form	Peak Energy (eV) 1s→π*	Peak Energy (eV) 1s→σ*
C=N in aromatic 6-membered ring (putative)	397.88	
C=N in aromatic 6-membered ring (1&2N)	398.76	408.01, 412.28
C=N in aromatic 6-membered oxygenated ring (2N)	399.20	405.00, 408.40
C=N in aromatic 5-membered ring (2N) and nitrile C≡N	400.05	406.43, 410.87, 413.75
Quaternary C-N in non-aromatic 6-membered ring	400.46	408.86
C-N in aromatic 6-membered oxygenated ring (2N)	400.64	406.92
C-N in non-aromatic 6-membered oxygenated ring (2N)	401.15	406.10
C-N in aromatic 5-membered ring (2N)	401.43	
C-N in aromatic 5-membered ring (1N)	402.40	407.00
C=N in aromatic 6-membered ring (1&2N, secondary peak)	402.51	
C-NH_2: aliphatic amine bonded to aromatic 6-membered ring	403.00	
C-NO_2: nitro bonded to aromatic 6-membered ring	403.65	412.79, 413.65
C=N in aromatic 6-membered ring (1&2N, secondary peak)	404.11	
N-H		405.00
C-NH_2: aliphatic amine		406.58

Peak assignments were compiled from spectra collected by the authors and by ref.[6]. Median values were calculated for N structures represented in more than one standard compound (Supplementary Table 3). The peak centered at 397.88 is not associated with a specific standard compound, but was identified by deconvolution of PyOM spectra and is closest in position to spectral features associated with aromatic 6-membered rings.

Supplementary Table 2. Gaussian curve areas associated with N bonds measured with N *K*-edge NEXAFS.

N Form	Peak Energy (eV)	Transition	PyOM	PyOM+NH_4^+	PyOM+NH_3
C=N in aromatic 6-membered ring (putative)	397.88	1*s*→π*	0.0196	0.0416	0.1943
C=N in aromatic 6-membered ring (1&2N)	398.76	1*s*→π*	0.0000	0.0000	0.1194
C=N in aromatic 6-membered oxygenated ring (2N)	399.20	1*s*→π*	0.0493	0.0000	0.1226
C≡N (nitrile) and C=N in aromatic 5-membered ring (2N)	400.05	1*s*→π*	0.0230	0.0968	0.3930
C-N in aromatic 6-membered oxygenated ring (2N)	400.64	1*s*→π*	0.0493	0.0000	0.1528
C-N in non-aromatic 6-membered oxygenated ring (2N)	401.15	1*s*→π*	0.0000	0.0000	0.1584
C-N in aromatic 5-membered ring (2N)	401.43	1*s*→π*	0.0847	0.1530	0.4950
C-N in aromatic 5-membered ring (1N)	402.40	1*s*→π*	0.0977	0.0000	0.4165
C-NH_2: aliphatic amine bonded to aromatic 6-membered ring	403.00	1*s*→π*	0.0000	0.0931	0.1906
C-NO_2: nitro bonded to aromatic 6-membered ring	403.65	1*s*→π*	0.1838	0.2091	0.3239
C=N in aromatic 6-membered ring (1&2N, secondary peak)	404.11	1*s*→π*	0.0000	0.0000	0.1907
N-H	405.00	1*s*→σ*	0.0000	0.0000	2.0880
C-NH_2: aliphatic amine	406.58	1*s*→σ*	3.1388	4.8523	0.0000
	408.01	1*s*→σ*	0.0000	0.0000	0.8896
	408.86	1*s*→σ*	0.0000	0.0000	0.4479
	410.87	1*s*→σ*	0.0000	0.5391	0.4743
	413.75	1*s*→σ*	0.2648	0.3503	1.7019

Deconvolution of NEXAFS spectra collected from PyOM, PyOM following exposure to NH_4^+, and PyOM following exposure to NH_3 was used to determine the area represented by different N bonds. Area fractions listed in the main text were only interpreted for π^* bonds because of the high degree of peak overlap in the σ^* region.

Supplementary Table 3. Major features of N *K*-edge NEXAFS spectra collected from N standard compounds.

Standard compound	1*s*→π*	hwhm	1*s*→π*	hwhm	1*s*→π*	hwhm	1*s*→σ*	hwhm	1s→σ*	hwhm
Acridine	398.28	0.51					407.89	1.54	412.28	2.92
2,3-Pyrazinedicaboxylic acid	398.72	0.33	399.48	0.84	402.42	0.49	407.60	2.41		
2,3-Pyridinedicaboxylic acid	398.79	0.32	400.25	0.41			403.88	0.47	408.12	1.24
2-Pyrimidinecarbonitrile[6]	398.80	0.40	400.00	0.40	402.60	0.40	404.50	2.00	408.60	2.00
Cytosine[6]	399.20	0.40	400.60	0.40	403.00	0.40	405.00	2.00	408.40	2.00
3,5-Pyrazoledicarboxylic acid	399.76	0.33	401.20	0.57			406.47	1.87	410.87	1.87
L-Histidine	400.05	0.28	401.43	0.51			406.38	2.01	413.85	2.66
4-Nitro-1H-pyrazole	400.27	0.40	401.88	0.50	403.75	0.45	413.65	1.89		
1-Ethyl-4-(methoxycarbonyl)-pyridinium iodide	400.46	0.38			404.11	0.35	408.86	2.27		
2-Pyridone	400.68	0.57					406.92	2.05		
Glycine anhydride	401.15	0.51					406.10	1.81	412.86	2.35
Carbazole[6]	402.40	0.40					407.00	2.00		
4-Nitro-phenylacetic acid	403.54	0.46					412.79	2.29		
Ammonium sulfate							405.82	1.47		
L-Isoleucine							406.58	2.36		

Peak centers and half widths at half maximum (hwhm) are shown in eV. Spectra were collected by the authors and by ref.[6] where indicated.

Supplementary Table 4. Integrated peak areas from ^{15}N-NMR spectrum of oxidized PyOM following exposure to gaseous [^{15}N]-NH_3.

Chemical Shift	Integral Center	Integral Area	Assignment
155.9	160.2	11.3	Heterocycles
107.1	111.3	30.8	Amides
20.3	20.6	58.0	NH_4^+ and Amines

Chemical shifts and integral centers are shown in ppm; assignments are taken from refs[7,8]. No NMR signal was detected in the CPMAS spectrum collected from oxidized PyOM following exposure to [^{15}N]-NH_4^+, so a SEDPMAS spectrum was not collected from this sample for quantitation. See Fig. 3c and Supplementary Fig. 14 for matching spectra.

Supplementary References

1. Knicker, H. Solid state CPMAS ^{13}C and ^{15}N NMR spectroscopy in organic geochemistry and how spin dynamics can either aggravate or improve spectra interpretation. *Org. Geochem.* **42**, 867-890 (2011).
2. Jaeger, R., Stohr, J. & Kendelewicz, T. X-ray induced electron stimulated desorption versus photon stimulated desorption: NH_3 on Ni(110). *Surf. Sci.* **134**, 547-565 (1983).
3. Parent, P. *et al*. The photoproduction of N_2 in condensed ammonia. Science of Solar System Ices: A Cross Disciplinary Workshop; 2008 May 5-8; Oxnard, USA. Lunar and Planetary Institute. https://www.lpi.usra.edu/meetings/scssi2008/pdf/9034.pdf (2008).
4. Wight, G. R. & Brion, C. E. K-shell excitation of CH_4, NH_3, H_2O, CH_3OH, CH_3OCH_3 and CH_3NH_2 by 2.5 keV electron impact. *J. Electron Spectrosc.* **4**, 25-42 (1974).
5. National Institute of Standards and Technology. NIST Chemistry WebBook. Available online at https://doi.org/10.18434/T4D303 (2016).
6. Leinweber, P. *et al*. Nitrogen K-edge XANES - An overview of reference compounds used to identify 'unknown' organic nitrogen in environmental samples. *J. Synchrotron Radiat.* **14**, 500-511 (2007).
7. Knicker, H. The feasibility of using DCPMAS ^{15}N ^{13}C NMR spectroscopy for a better characterization of immobilized ^{15}N during incubation of ^{13}C- and ^{15}N-enriched plant material. *Org. Geochem.* **33**, 237–246 (2002).
8. Smernik, R. J. & Baldock, J. A. Solid-state ^{15}N NMR analysis of highly ^{15}N-enriched plant materials. *Plant Soil* **275**, 271-283 (2005).
